# A comparative study of eradication rates, adverse events, and compliance between triple therapy and bismuth-containing quadruple therapy in children with Helicobacter pylori infection

**DOI:** 10.3389/fped.2026.1819833

**Published:** 2026-04-22

**Authors:** Yonghao Qian, Xiaozhen Hua

**Affiliations:** 1 Department of General Pediatrics, The People’s Hospital of Taishun County, Wenzhou, China; 2 Department of Pediatrics, The People’s Hospital of Cangnan, Cangnan Hospital of Wenzhou Medical University, Wenzhou, China

**Keywords:** bismuth-containing quadruple therapy, children, eradication rate, Helicobacter pylori, symptom assessment, triple therapy

## Abstract

**Purpose:**

To compare the eradication efficacy, symptom improvement, and safety between standard triple therapy and bismuth-containing quadruple therapy for Helicobacter pylori (*H. pylori*) infection in children.

**Methods:**

This retrospective cohort study included 161 pediatric patients who completed either a 14-day triple therapy (proton pump inhibitor, clarithromycin, amoxicillin; *n* = 78) or a 14-day bismuth-containing quadruple therapy (adding colloidal bismuth subcitrate; *n* = 83). The primary outcome was the eradication rate assessed by ^13^C-urea breath test 4 weeks post-treatment. Secondary outcomes included changes in abdominal pain, bloating, and nausea scores (measured by a 4-point Likert scale before and after treatment) and the incidence of adverse events.

**Results:**

The eradication rate was significantly higher in the bismuth-containing quadruple therapy group (90.36%) compared to the triple therapy group (74.36%) (*P* = 0.007). Post-treatment symptom scores improved more markedly with bismuth-containing quadruple therapy for abdominal pain (0.68 vs. 0.85, *P* < 0.001), bloating (0.55 vs. 0.64, *P* = 0.021), and nausea (0.35 vs. 0.41, *P* = 0.024). In a subgroup analysis restricted to patients with successful eradication, the bismuth-containing quadruple therapy group still had significantly lower abdominal pain scores (*P* = 0.002), suggesting a potential independent effect of bismuth on abdominal pain relief. The incidence of adverse events and the medication compliance rate were comparable between the two groups (*P* > 0.05). Multivariate analysis identified bismuth-containing quadruple therapy as a protective factor against eradication failure (Adjusted OR = 0.351, *P* = 0.018).

**Conclusion:**

Bismuth-containing quadruple therapy is potentially advantageous to standard triple therapy for eradicating *H. pylori* and alleviating associated symptoms in children, with a similar safety and compliance profile.

## 1 Introduction

Helicobacter pylori (*H. pylori*) infection is a prevalent condition affecting millions of individuals worldwide, including children (1). This bacterium is a major cause of chronic gastritis, peptic ulcer disease, and gastric cancer. In pediatric populations, *H. pylori* infection can lead to significant morbidity, impacting growth, nutrition, and overall quality of life. Effective eradication of *H. pylori* is crucial for preventing these complications and improving long-term health outcomes (2, 3). Traditional treatment regimens, such as triple therapy consisting of a proton pump inhibitor (PPI), clarithromycin, and amoxicillin, have been widely used but are increasingly challenged by rising rates of antibiotic resistance, leading to suboptimal eradication rates. Consequently, there is a pressing need to explore alternative therapeutic strategies that can overcome these limitations (4–6).

Bismuth-containing quadruple therapy has emerged as a promising alternative to standard triple therapy. This regimen typically includes a PPI, bismuth subsalicylate or bismuth subcitrate, and two antibiotics, often clarithromycin and amoxicillin. The inclusion of bismuth offers several potential advantages. Bismuth compounds possess antimicrobial properties that can inhibit bacterial protein synthesis, disrupt cell membranes, and reduce bacterial adherence to the gastric mucosa. These mechanisms complement the actions of conventional antibiotics, potentially enhancing bacterial eradication. Bismuth may help mitigate the effects of antibiotic resistance by providing an alternative pathway for eradicating *H. pylori* (7–9).

The management of *H. pylori* infection in children presents unique challenges due to differences in physiology, immune response, and medication metabolism compared to adults. Pediatric patients may also experience different symptom profiles and require tailored treatment approaches. Ensuring high eradication rates is particularly important in children to prevent the development of severe complications later in life (10, 11). Achieving these rates with current therapies remains challenging. Triple therapy, while effective in many cases, faces increasing resistance issues, leading to lower success rates. The addition of bismuth to quadruple therapy could address some of these limitations by enhancing bacterial clearance and reducing recurrence (12, 13). Moreover, the tolerability and compliance of pediatric patients must be considered when selecting treatment options. High compliance is critical for successful eradication, and any new regimen should not impose additional burdens on patients or caregivers.

Comparative studies evaluating the efficacy, safety, and compliance of different *H. pylori* treatment regimens in pediatric populations are limited. Most existing data come from adult studies, which may not be directly applicable to children due to differences in pathophysiology and response to therapy. Although bismuth-containing quadruple therapy has been compared with triple therapy in adults, pediatric-specific data remain scarce. A comprehensive evaluation of bismuth-containing quadruple therapy vs. standard triple therapy in children is necessary to guide clinical decision-making. Such a study would provide valuable insights into the relative merits of each approach, helping clinicians choose the most appropriate treatment for their pediatric patients. By addressing these gaps in knowledge, future research can contribute to better patient outcomes and more effective management of *H. pylori* infections in children. This study aims to fill this gap by comparing the eradication rates, adverse events, and compliance between triple therapy and bismuth-containing quadruple therapy in pediatric patients with *H. pylori* infection, thereby informing optimal therapeutic strategies for this vulnerable population.

## 2 Materials and methods

### 2.1 Study design and participants

This retrospective cohort study was carried out in the Pediatrics Department of the People's Hospital of Cangnan (Approval No. 2024027). The Hospital Ethics Committee reviewed and approved the study protocol. Given the retrospective design of the research, the committee waived the need for obtaining informed consent. Electronic medical records were searched to identify pediatric patients diagnosed with chronic gastritis and confirmed *H. pylori* infection between January, 2020, and December, 2023. The diagnosis of *H. pylori* infection was based on positive results from either rapid urease test (RUT) or histopathological examination of gastric mucosal biopsies obtained during upper gastrointestinal endoscopy (14). All enrolled patients underwent upper gastrointestinal endoscopy as part of the diagnostic workup, and biopsy specimens were obtained for RUT and histopathological examination. In our center, all RUT-positive cases were confirmed by histopathological examination to ensure diagnostic reliability and to minimize the risk of false-positive results.

Inclusion criteria were: (1) age between 4 and 17 years; (2) confirmed *H. pylori*-positive chronic gastritis (14); (3) completion of either 14-day standard triple therapy or 14-day bismuth-containing quadruple therapy as the first-line eradication regimen; (4) availability of a follow-up ^13^C-urea breath test (UBT) result at 4 weeks after treatment completion. Exclusion criteria included: (1) previous history of *H. pylori* eradication therapy; (2) incomplete medical records or loss to follow-up; (3) use of antibiotics, proton pump inhibitors (PPIs), H2-receptor antagonists, or bismuth preparations within the 4 weeks prior to the initial diagnosis; (4) known allergy to any of the study medications; (5) presence of severe systemic diseases, gastrointestinal surgery history, or other chronic conditions that could interfere with the assessment.

A total of 230 medical records were initially screened. Sixty-nine patients were excluded for the following reasons: previous *H. pylori* eradication therapy (*n* = 20), use of antibiotics, PPIs, H2-receptor antagonists, or bismuth within 4 weeks before diagnosis (*n* = 15), incomplete medical records or loss to follow-up (*n* = 25), known allergy to study medications (*n* = 5), and severe systemic diseases or gastrointestinal surgery (*n* = 4). Thus, 161 patients were included in the final analysis. All endoscopic examinations were performed in accordance with the indications recommended by the ESPGHAN/NASPGHAN joint guidelines for the management of *H. pylori* infection in children (14).

### 2.2 Treatment regimens

Patients were naturally allocated into two groups based on the treatment they actually received as documented in their medical records. The specific medication doses were adjusted according to body weight following standard pediatric guidelines. All medications were administered orally.

Triple Therapy Group: Patients received a 14-day regimen consisting of a PPI + clarithromycin + amoxicillin. The dosages were: Amoxicillin (manufacturer: North China Pharmaceutical Group Co., Ltd., China; specification: 0.25 g per capsule) at 50 mg/(kg·day), divided into two doses per day (maximum dose 1 g, twice daily); Clarithromycin (manufacturer: Livzon Pharmaceutical Group Inc., China; specification: 0.25 g per tablet) at 15–20 mg/(kg·day), divided into two doses per day (maximum dose 0.5 g, twice daily); Omeprazole (manufacturer: AstraZeneca PLC, UK, or domestic generic equivalent; specification: 20 mg per capsule) at 0.6–1.0 mg/(kg·day), divided into two doses per day, administered before meals (maximum dose 20 mg, twice daily).

Bismuth-containing Quadruple Therapy Group: Patients received a 14-day regimen consisting of a PPI +amoxicillin + clarithromycin + Colloidal Bismuth Subcitrate. The dosages for amoxicillin, clarithromycin, and omeprazole were identical to the Triple Therapy Group. Additionally, Colloidal Bismuth Subcitrate (manufacturer: Livzon Pharmaceutical Group Inc., China; common brand: Livzon Dele; specification: 110 mg per capsule) was administered at 6–8 mg/(kg·day) for children older than 6 years, divided into two doses per day taken before meals (maximum dose 220 mg, twice daily).

### 2.3 Efficacy assessment

The primary endpoint was the rate of successful *H. pylori* eradication, which was determined using a negative urea breath test result 4 weeks following the conclusion of antibiotic treatment. The UBT was performed using a dedicated infrared isotope analyzer (model: HY-IREX, manufacturer: Huayi Medical, China). A baseline sample of exhaled breath was obtained following an overnight fast. Patients then ingested 75 mg of ^13^C-urea powder (Beijing Zhibo Union, China) dissolved in 50 mL of water. A second breath sample was collected 30 min later. The change in the ^13^CO_2_/^12^CO_2_ ratio (*δ*-value) was measured. A *δ*-value of less than 4‰ was considered indicative of successful *H. pylori* eradication. All patients had unequivocal results (either <4‰ or ≥4‰); no borderline cases were encountered.

### 2.4 Symptom assessment

Clinical symptom improvement was a secondary outcome. The severity of three core symptoms—abdominal pain, bloating, and nausea—was evaluated using a standardized four-point Likert scale (0, none; 1, mild; 2, moderate; and 3, severe) recorded during clinic visits before treatment and at the 4-week follow-up visit. These scores were extracted from standardized clinical records, as symptom assessment using this scale is part of the routine clinical practice in our department. The time to symptom relief (in days) for each symptom was also extracted from the patients' symptom diaries or clinical progress notes, defined as the first day the patient reported the symptom as absent or mild (score 0 or 1) after starting treatment. This scale has been specifically applied to assess symptoms in Chinese children with H. pylori-associated gastritis (15). To further support the methodological robustness of our symptom assessment, we also refer to validated pediatric gastrointestinal symptom scales reported in the international literature (16).

### 2.5 Laboratory parameter assessment

Routine laboratory tests performed as part of standard clinical care before and after treatment were analyzed. Venous blood samples were collected after an 8-hour fast. Complete blood count, including hemoglobin and platelet count, was measured using an automated hematology analyzer (model: BC-6800 Plus, manufacturer: Mindray, China). C-reactive protein level was determined using a latex-enhanced immunoturbidimetric assay on a clinical chemistry analyzer (model: Cobas c 503, manufacturer: Roche Diagnostics, Switzerland).

### 2.6 Assessment of adverse events and compliance

Adverse events (AEs) occurring during the treatment period were systematically recorded from clinical notes and patient-reported symptom diaries. Events such as nausea, abdominal discomfort/pain, diarrhea, taste disturbance/bitter mouth, skin rash, and vomiting were specifically documented. Medication compliance was calculated based on pharmacy dispensing records and patient/parental reporting at the follow-up visit. Compliance rate (%) was determined as (number of doses actually taken/number of doses prescribed) × 100%. Patients were categorized as having “Good” compliance (≥90%), “Fair” compliance (80%–89%), or “Poor” compliance (<80%).

### 2.7 Statistical analysis

We performed statistical analysis in SPSS 29.0 (IBM Corp., USA). For group comparisons, normally distributed continuous variables (mean ± SD) were analyzed with the independent-samples *t*-test. For comparisons of symptom scores between the two treatment groups post-treatment, independent-samples *t*-test was used. For within-group comparisons of symptom scores before and after treatment, paired *t*-test was applied. Categorical variables (frequency, percentage) were analyzed with the Chi-square or Fisher's exact test. Significance was defined as two-tailed *P* < 0.05. Potential factors for eradication failure (univariate *P* < 0.1 or clinical relevance) were examined using multivariable logistic regression, yielding adjusted odds ratios (ORs) with 95% confidence intervals (CIs). As all included patients completed the full treatment course and had available follow-up UBT results, the analysis was performed on a per-protocol basis. No intention-to-treat analysis was required because there were no missing outcome data.

To assess whether temporal changes in prescribing practices influenced the results, we added year of treatment (categorized as 2020–2021 vs. 2022–2023) as an additional covariate to the simplified multivariate model. The adjusted odds ratio for bismuth-containing quadruple therapy remained consistent (Adjusted OR = 0.371, 95% CI: 0.158–0.882, *P* = 0.025), and year of treatment was not independently associated with eradication failure (*P* = 0.532). These findings suggest that the protective effect of bismuth-containing quadruple therapy was robust to adjustment for temporal trends.

Propensity score matching was considered as a method to address potential selection bias. However, given the relatively small sample size (*n* = 161) and the limited number of treatment failure events (*n* = 28), applying PSM would have substantially reduced the effective sample size and compromised statistical power. Moreover, baseline characteristics between the two treatment groups were well balanced across all measured covariates, suggesting that major observed confounders were comparable. We elected to present the unmatched analysis with multivariate adjustment for key clinical covariates, and we acknowledge the potential for residual confounding as a limitation.

## 3 Results

### 3.1 Study population and baseline characteristics

The baseline demographic and clinical characteristics of the 161 enrolled patients are summarized in Table 1. The Triple Therapy group (*n* = 78) and the Bismuth-containing Quadruple Therapy group (*n* = 83) were well-matched, with no significant differences in age, sex distribution, body weight, symptom duration, or the prevalence of recurrent vomiting, peptic ulcer disease, and moderate-to-severe gastritis at endoscopy (all *P* > 0.05).

**Table 1:** Baseline demographic and clinical characteristics.

Parameters	Triple therapy group (*n* = 78)	Bismuth quadruple therapy group (*n* = 83)	*t*/*χ*^2^	*P*
Age (years)	10.24 ± 3.15	10.55 ± 2.89	0.658	0.511
Sex (male), *n* (%)	42 (53.85%)/36 (46.15%)	45 (54.22%)/38 (45.78%)	0.002	0.962
Body weight (kg)	37.62 ± 9.24	38.81 ± 9.72	0.793	0.429
Symptom duration (months)	7.35 ± 2.21	6.98 ± 2.87	0.932	0.353
Recurrent vomiting, *n* (%)	15 (19.23%)/63 (80.77%)	18 (21.69%)/65 (78.31%)	0.149	0.700
Peptic ulcer disease, *n* (%)	9 (11.54%)/69 (88.46%)	12 (14.46%)/71 (85.54%)	0.302	0.583
Endoscopic findings (gastritis grade: mild/moderate to severe), *n* (%)	45 (57.69%)/33 (42.31%)	52 (62.65%)/31 (37.35%)	0.413	0.521

### 3.2 Eradication rates

As presented in Table 2, the *H. pylori* eradication rate was significantly higher in the Bismuth-containing Quadruple Therapy group (90.36%) compared to the Triple Therapy group (74.36%) (*χ*² = 7.167, *P* = 0.007).

**Table 2:** *H. pylori* eradication outcomes at 4 weeks post-treatment.

Parameters	Triple therapy group (*n* = 78)	Bismuth quadruple therapy group (*n* = 83)	*χ* ^2^	*P*
Eradication success, *n* (%)	58 (74.36%)	75 (90.36%)	7.167	0.007
Negative conversion	58	75		
Non-negative conversion	20	8		

### 3.3 Symptom improvement

**Figure 1:**
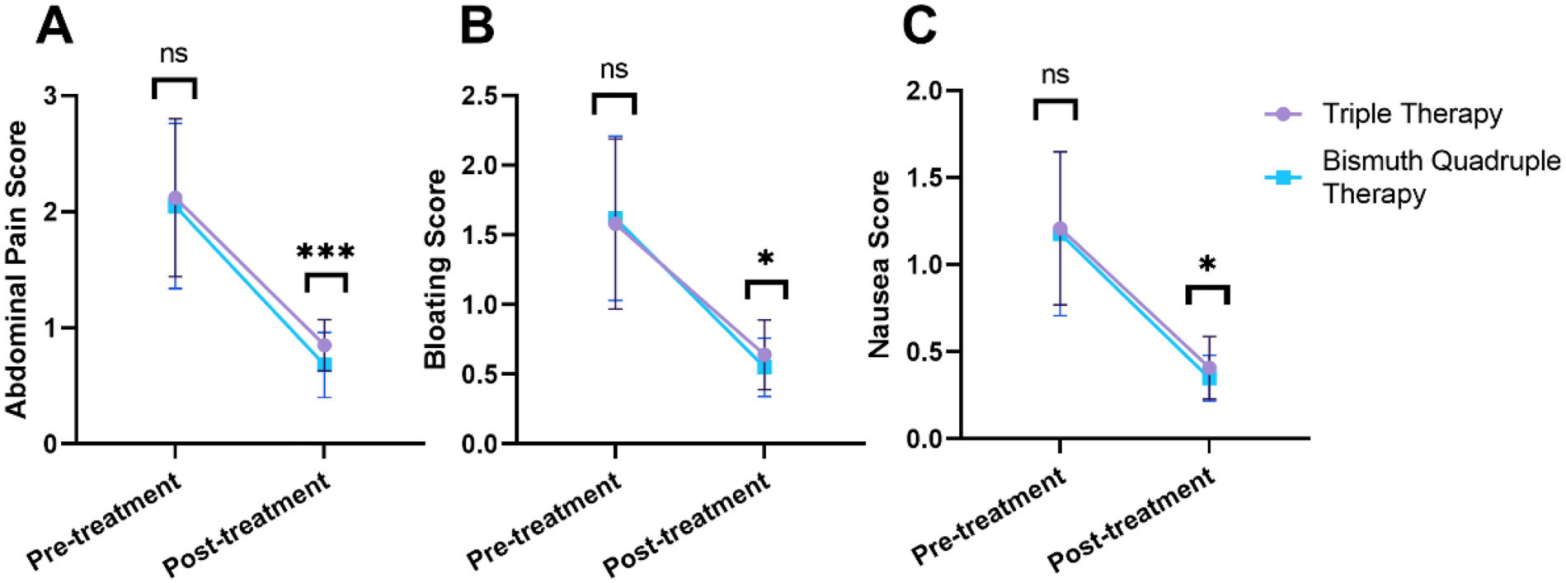
Improvement in clinical symptom scores after treatment. **(A)** Abdominal pain score; **(B)** bloating score; **(C)** nausea score. ns, no significant difference; *: *P* < 0.05; ***: *P* < 0.001. Comparisons between the two groups were performed using independent-samples *t*-test.

The changes in clinical symptom scores are detailed in Figure 1. At baseline, the severity of abdominal pain, bloating, and nausea was comparable between the Triple Therapy group and the Bismuth-containing Quadruple Therapy group (all *P* > 0.05). Following treatment, the post-treatment abdominal pain score was significantly lower in the Bismuth-containing Quadruple Therapy group (0.68 ± 0.28) than in the Triple Therapy group (0.85 ± 0.22) (*P* < 0.001). Similarly, greater reductions were observed in the Bismuth-containing Quadruple Therapy group for both bloating score (0.55 ± 0.21 vs. 0.64 ± 0.25, *P* = 0.021) and nausea score (0.35 ± 0.13 vs. 0.41 ± 0.18, *P* = 0.024). It should be noted that the absolute differences in symptom scores between the two treatment groups were numerically small. While statistically significant, the clinical relevance of these small differences requires further validation.

**Figure 2:**
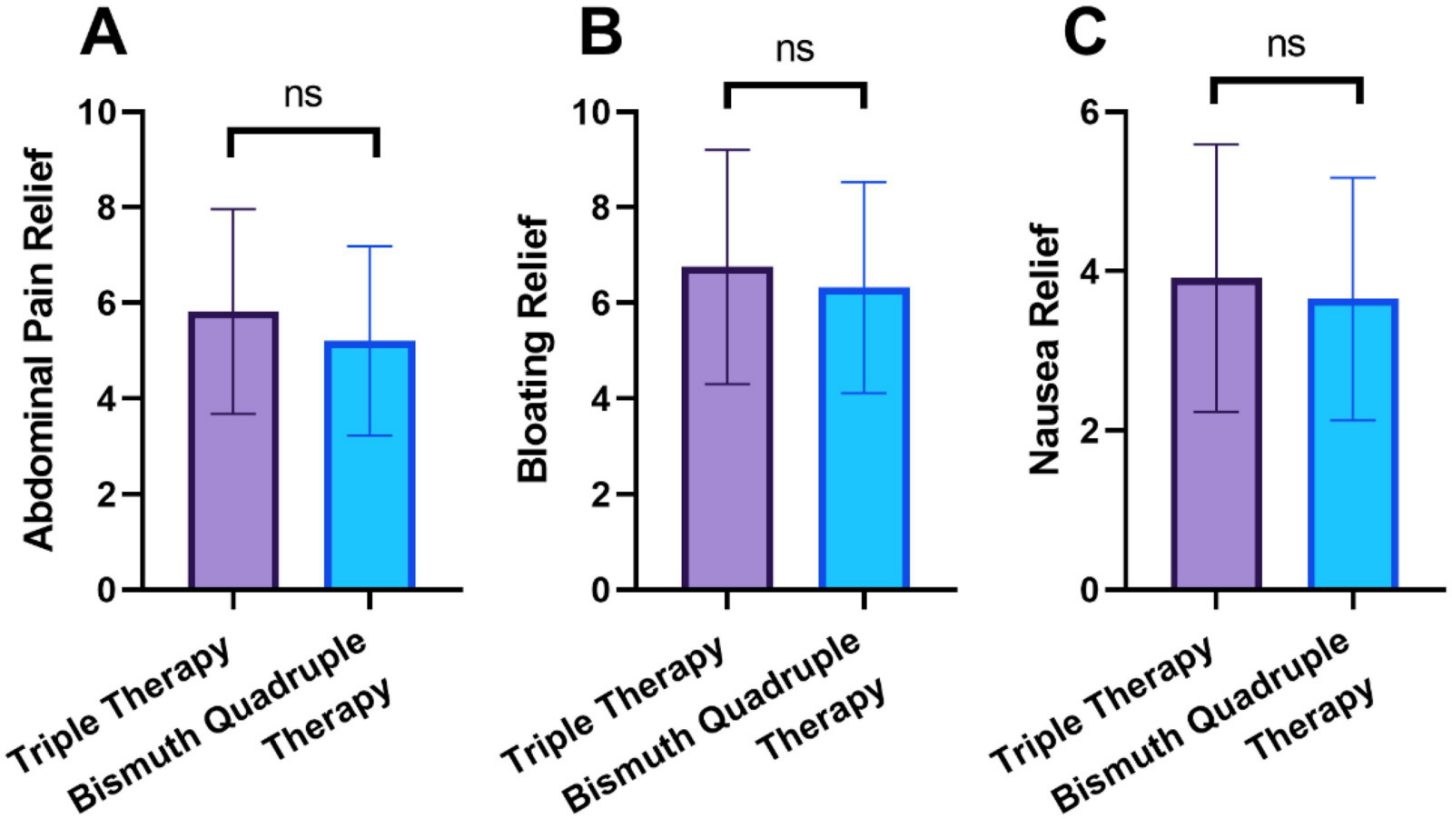
Time to symptom relief after initiation of therapy (days). **(A)** Abdominal pain relief; **(B)** bloating relief; **(C)** nausea relief. ns, no significant difference.

The time required for symptom relief after initiating therapy is shown in Figure 2. The mean time to abdominal pain relief was 5.21 ± 1.98 days in the Bismuth-containing Quadruple Therapy group and 5.82 ± 2.14 days in the Triple Therapy group (*P* = 0.063). The times to bloating relief and nausea relief were also similar between the groups (*P* > 0.05).

To further explore the relationship between symptom improvement and treatment success, we compared post-treatment symptom scores between patients with successful *H. pylori* eradication (*n* = 133) and those with eradication failure (*n* = 28), regardless of the treatment regimen received. Patients with successful eradication had significantly lower scores for abdominal pain (0.61 ± 0.23 vs. 1.08 ± 0.31, *P* < 0.001), bloating (0.52 ± 0.20 vs. 0.79 ± 0.28, *P* < 0.001), and nausea (0.31 ± 0.12 vs. 0.54 ± 0.19, *P* < 0.001) compared with those in whom eradication failed (Table 3).

**Table 3:** Post-treatment symptom scores according to eradication outcome.

Parameters	Eradication success (*n* = 133)	Eradication failure (*n* = 28)	*t*	*P*
Abdominal pain score	0.61 ± 0.23	1.08 ± 0.31	7.736	<0.001
Bloating score	0.52 ± 0.2	0.79 ± 0.28	4.899	<0.001
Nausea score	0.31 ± 0.12	0.54 ± 0.19	6.139	<0.001

To determine whether bismuth-containing quadruple therapy has any symptom-alleviating effects beyond those attributable to successful eradication alone, we further compared post-treatment symptom scores between the two treatment groups restricted to patients who achieved successful eradication (*n* = 133). As shown in Table 4, among patients with successful eradication, the bismuth-containing quadruple therapy group still had significantly lower abdominal pain scores compared with the triple therapy group (0.64 ± 0.22 vs. 0.77 ± 0.24, *P* = 0.002). However, no significant differences were observed for bloating (*P* = 0.129) or nausea (*P* = 0.158). These findings suggest that while the symptom improvement in the bismuth-containing quadruple therapy group is largely driven by its higher eradication rate, bismuth may have an additional independent effect on abdominal pain relief, possibly through its local mucosal protective or mild anti-inflammatory properties.

**Table 4:** Post-treatment symptom scores in patients with successful eradication, by treatment group.

Parameters	Triple therapy success (*n* = 58)	Bismuth quadruple therapy success (*n* = 75)	*t*	*P*
Abdominal pain score	0.77 ± 0.24	0.64 ± 0.22	3.171	0.002
Bloating score	0.55 ± 0.21	0.49 ± 0.19	1.53	0.129
Nausea score	0.33 ± 0.13	0.30 ± 0.11	1.42	0.158

### 3.4 Laboratory parameters

Serial measurements of key laboratory parameters are shown in Table 5. Levels of hemoglobin, platelet count, and C-reactive protein remained stable throughout the treatment period in both groups, with no significant within-group changes or between-group differences observed post-treatment (all *P* > 0.05).

**Table 5:** Changes in key laboratory parameters after treatment.

Parameters	Triple therapy group (*n* = 78)	Bismuth quadruple therapy group (*n* = 83)	*t*	*P*
Hemoglobin (g/L)				
Pre-treatment	128.51 ± 10.32	126.81 ± 11.12	1.004	0.317
Post-treatment	129.08 ± 9.81	127.52 ± 10.62	0.961	0.338
Platelet count (×10⁹/L)				
Pre-treatment	285.34 ± 58.21	278.95 ± 62.14	0.673	0.502
Post-treatment	280.15 ± 55.43	275.82 ± 60.58	0.473	0.637
C-reactive protein (mg/L)				
Pre-treatment	3.25 ± 1.11	3.41 ± 1.34	0.814	0.417
Post-treatment	2.88 ± 1.05	2.79 ± 1.07	0.558	0.578

### 3.5 Adverse events

The spectrum and incidence of adverse events recorded during treatment are listed in Table 6. The occurrence of nausea, abdominal discomfort, diarrhea, taste disturbance, skin rash, and vomiting did not differ significantly between the Triple Therapy and the Bismuth-containing Quadruple Therapy groups (all *P* > 0.05).

**Table 6:** Incidence and spectrum of adverse events during treatment.

Parameters	Triple therapy group (*n* = 78)	Bismuth quadruple therapy group (*n* = 83)	*χ* ^2^	*P*
Nausea	5 (6.41%)	12 (14.46%)	2.758	0.097
Abdominal discomfort/pain	7 (8.97%)	11 (13.25%)	0.741	0.389
Diarrhea	8 (10.26%)	4 (4.82%)	1.723	0.189
Taste disturbance/bitter mouth	3 (3.85%)	9 (10.84%)	2.854	0.091
Skin rash	2 (2.56%)	1 (1.2%)	0.003	0.957
Vomiting	1 (1.28%)	3 (3.61%)	0.197	0.657

### 3.6 Medication compliance

Assessment of medication adherence is presented in Table 7. Both groups demonstrated high compliance, with the proportion of patients achieving good compliance (≥90%) and the mean compliance rate being similar between the Triple Therapy group and the Bismuth-containing Quadruple Therapy group (*P* > 0.05).

**Table 7:** Medication compliance assessment.

Parameters	Triple therapy group (*n* = 78)	Bismuth quadruple therapy group (*n* = 83)	*χ* ^2^	*P*
Good (≥90% doses taken), *n* (%)	65 (83.33%)	65 (78.31%)	0.846	0.655
Fair (80%–89% doses taken), *n* (%)	10 (12.82%)	15 (18.07%)		
Poor (<80% doses taken), *n* (%)	3 (3.85%)	3 (3.61%)		
Mean compliance rate (%)	92.15 ± 3.04	91.87 ± 3.12	0.569	0.570

### 3.7 Univariate and multivariate analysis

Univariate analysis was first performed to identify factors associated with H. pylori eradication failure (Table 8). Among the variables examined, treatment group (bismuth-containing quadruple therapy vs. triple therapy), medication compliance (poor vs. good/fair), and endoscopic gastritis grade (moderate-to-severe vs. mild) were significantly associated with eradication failure (all *P* < 0.05). Age and symptom duration were not significantly associated (*P* > 0.05).

**Table 8:** Univariate logistic regression analysis of factors associated with *H. pylori* eradication failure.

Parameters	Adjusted odds ratio (OR)	95% Confidence interval	*P*
Treatment group (bismuth quadruple vs. triple)	0.310	0.126–0.762	0.011
Poor medication compliance (<80%)	10.892	1.889–63.216	0.008
Moderate-to-severe gastritis at endoscopy	3.836	1.662–8.849	0.002
Age	0.993	0.878–1.136	0.865
Symptom duration	1.056	0.901–1.226	0.543

Multivariate logistic regression analysis identified independent factors associated with eradication failure (Table 9). Receiving Bismuth-containing Quadruple Therapy was a protective factor against failure, associated with a significantly lower risk of failure compared to Triple Therapy (Adjusted OR = 0.351, 95% CI: 0.148–0.835, *P* = 0.018). Poor medication compliance (<80%) (Adjusted OR = 4.256, 95% CI: 1.512–11.978, *P* = 0.006) and the presence of moderate-to-severe gastritis at baseline endoscopy (Adjusted OR = 2.954, 95% CI: 1.256–6.948, *P* = 0.013) were identified as significant risk factors for eradication failure.

**Table 9:** Multivariate logistic regression analysis of factors associated with *H. pylori* eradication failure.

Parameters	Adjusted odds ratio (OR)	95% Confidence interval	*P*
Treatment group (bismuth quadruple vs. triple)	0.351	0.148–0.835	0.018
Poor medication compliance (<80%)	4.256	1.512–11.978	0.006
Moderate-to-severe gastritis at endoscopy	2.954	1.256–6.948	0.013

## 4 Discussion

The management of *H. pylori* infection in pediatric patients remains a significant challenge due to the rising rates of antibiotic resistance and the need for safe, effective treatment options. This study aimed to compare the eradication rates, symptom improvement, adverse events, and medication compliance between triple therapy and bismuth-containing quadruple therapy in children diagnosed with chronic gastritis and confirmed *H. pylori* infection.

One of the primary goals of treating *H. pylori* infection is achieving high eradication rates to prevent complications such as peptic ulcers and gastric cancer. In this study, the bismuth-containing quadruple therapy group demonstrated higher eradication rates compared to the triple therapy group. This enhanced efficacy could be attributed to the additional use of bismuth, which has multiple mechanisms of action. Bismuth compounds can inhibit bacterial protein synthesis, disrupt cell membranes, and reduce bacterial adherence to gastric mucosa. Bismuth ions can form complexes with bacterial proteins, leading to the disruption of essential metabolic pathways within the bacteria. Bismuth's ability to bind to and neutralize toxins produced by *H. pylori* may further enhance its antibacterial activity (17–19). These effects, combined with the standard antibiotics used in both therapies, may contribute to more effective bacterial clearance. Furthermore, the inclusion of bismuth may help overcome resistance to clarithromycin and amoxicillin, which are common issues with triple therapy alone (20, 21). By providing an alternative pathway for bacterial eradication, bismuth-containing quadruple therapy offers a more robust approach to managing *H. pylori* infections in children. Previous studies have also explored the efficacy of bismuth-containing quadruple therapy in managing *H. pylori* infections. For instance, a study by Miao et al. (22) found similar trends as our study in eradication rates when comparing bismuth-containing quadruple therapy, sequential therapy to standard triple therapy.

Improvement in clinical symptoms is another critical outcome measure in assessing the effectiveness of *H. pylori* treatments. Post-treatment assessments revealed greater reductions in abdominal pain, bloating, and nausea scores in the bismuth-containing quadruple therapy group compared to the triple therapy group. This difference in symptom relief could be linked to the higher eradication rates observed in the bismuth-containing quadruple therapy group. Effective bacterial eradication leads to reduced inflammation and irritation of the gastric mucosa, resulting in faster and more pronounced symptom improvement. The anti-inflammatory properties of bismuth may also play a role in alleviating gastrointestinal symptoms (23–25).

To understand whether this difference is solely due to the higher eradication rate of bismuth-containing quadruple therapy, we performed a subgroup analysis comparing symptom scores between the two treatment groups restricted to patients who achieved successful eradication. Interestingly, among patients with successful eradication, the bismuth-containing quadruple therapy group still had significantly lower abdominal pain scores than the triple therapy group, while bloating and nausea scores did not differ significantly. This finding suggests that bismuth may have an additional independent effect on abdominal pain relief beyond its contribution to higher eradication rates. Possible mechanisms include bismuth's local mucosal protective properties, its mild anti-inflammatory effects, or its ability to modulate gastric sensitivity (26–28). For bloating and nausea, however, symptom improvement appears to be primarily driven by successful bacterial eradication itself.

The incidence of adverse events did not differ significantly between the two treatment groups. Common adverse events such as nausea, abdominal discomfort, diarrhea, taste disturbance, skin rash, and vomiting were reported at similar frequencies in both groups. This similarity suggests that the addition of bismuth to quadruple therapy does not substantially increase the risk of adverse reactions. Bismuth is generally well-tolerated in pediatric populations, with minimal systemic absorption and primarily localized effects on the gastrointestinal tract. The absence of increased adverse events supports the safety profile of bismuth-containing quadruple therapy, making it a viable alternative to triple therapy without compromising patient safety (29, 30).

High medication compliance is essential for ensuring the success of any therapeutic regimen. In this study, both treatment groups demonstrated comparable levels of medication adherence, indicating that neither therapy posed significant barriers to compliance. The simplicity of the 14-day regimen and the manageable side effect profile likely contributed to the high adherence rates observed in both groups (7, 31). Importantly, the addition of bismuth to quadruple therapy did not appear to negatively impact compliance, suggesting that the extra medication component was well-accepted by pediatric patients. Ensuring good compliance is crucial for maximizing treatment efficacy, and these findings support the feasibility of using bismuth-containing quadruple therapy in routine clinical practice (32, 33).

While this study provides valuable insights into the comparative efficacy of triple therapy and bismuth-containing quadruple therapy, it has several limitations. As a retrospective cohort study, it is inherently subject to selection bias and uncontrolled confounding, and the level of evidence is lower than that of randomized controlled trials. Nevertheless, given the practical challenges of conducting randomized trials in pediatric *H. pylori* treatment, well-designed retrospective studies can provide valuable real-world evidence. Treatment allocation was non-randomized and based on physicians’ prescribing practices, introducing the potential for selection bias. Although baseline characteristics were well balanced between the two groups, unmeasured confounding factors—such as subtle differences in disease severity not captured by our data, temporal changes in prescribing practices, and regional variations in antibiotic resistance—may have influenced the results. To explore the potential impact of temporal trends, we performed a sensitivity analysis adding year of treatment to the multivariate model; the protective effect of bismuth-containing quadruple therapy remained consistent, and year of treatment was not independently associated with eradication failure. Nevertheless, residual confounding cannot be entirely excluded. We cannot rule out the possibility that clinicians preferentially prescribed bismuth-containing quadruple therapy to children with perceived higher risk of treatment failure (e.g., those with more severe endoscopic findings or a history of prior antibiotic use, although the latter was an exclusion criterion). This confounding by indication could bias the results in favor of the bismuth-containing regimen. The relatively small sample size and single-center retrospective design may limit the generalizability of the results. Propensity score matching was not performed due to the small sample size and limited number of failure events, which would have precluded meaningful matched analysis. Prospective, multicenter trials with larger patient populations are warranted to validate our findings and to establish the optimal duration and dosing of bismuth-containing quadruple therapy in children. Further research should explore the optimal duration and dosing of bismuth-containing quadruple therapy to maximize efficacy while minimizing adverse events. The absolute differences in symptom scores between the two groups were modest. Although these differences reached statistical significance, their clinical meaningfulness for pediatric patients and families should be interpreted with caution. Future studies should incorporate patient-reported outcome measures with established minimal clinically important differences (MCID) to better assess the real-world impact of symptom improvement. Additionally, clarithromycin susceptibility data were not available for the included patients, as antimicrobial susceptibility testing is not routinely performed for pediatric H. pylori infection in our center. Given the high prevalence of clarithromycin resistance in children in China, the lack of resistance data represents an important limitation. It remains possible that differential distribution of clarithromycin-resistant strains between the two treatment groups contributed to the observed difference in eradication rates, independent of the addition of bismuth.

In summary, this study demonstrates that bismuth-containing quadruple therapy achieves higher *H. pylori* eradication rates and greater symptom relief than standard triple therapy in children, with comparable safety and compliance. These findings support its use as a first-line option. However, the limitations of our retrospective design and modest sample size underscore the need for larger prospective trials to confirm these results and to optimize treatment protocols.

## 5 Conclusion

This study suggests that bismuth-containing quadruple therapy may offer potential advantages over standard triple therapy in the treatment of *H. pylori* infection in pediatric patients. It appears to enhance *H. pylori* eradication and alleviate associated symptoms, particularly abdominal pain, with a potential independent effect. For bloating and nausea, symptom improvement is largely attributable to successful eradication. Importantly, the addition of bismuth does not seem to increase the incidence of adverse events or negatively impact medication compliance compared to triple therapy. These findings indicate that bismuth-containing quadruple therapy could be a viable alternative for managing *H. pylori* infections in children, offering improved therapeutic outcomes without compromising safety or adherence.

## Data Availability

The raw data supporting the conclusions of this article will be made available by the authors, without undue reservation.
